# Impact and Cost-effectiveness of Regular Self-digital Anorectal Examination on Syphilis Among Gay, Bisexual, and Other Men Who Have Sex With Men: A Mathematical Modeling Study

**DOI:** 10.1093/infdis/jiaf310

**Published:** 2025-07-15

**Authors:** Hao Lai, Christopher K Fairley, Rui Li, Marcus Y Chen, Eric P F Chow, Basil Donovan, Denton Callander, Rebecca Guy, Julien Tran, Ei T Aung, Mingwang Shen, Lei Zhang

**Affiliations:** Phase I Clinical Trial Research Ward, The Second Affiliated Hospital of Xi’an Jiaotong University, Xi'an, China; China-Australia Joint Research Center for Infectious Diseases, School of Public Health, Xi’an Jiaotong University Health Science Center, China; Melbourne Sexual Health Centre, Alfred Health, Australia; Melbourne Sexual Health Centre, Alfred Health, Australia; School of Translational Medicine, Faculty of Medicine, Nursing and Health Science, Monash University, Melbourne, Australia; Heidelberg Institute of Global Health, Faculty of Medicine and University Hospital, Heidelberg University, Germany; Melbourne Sexual Health Centre, Alfred Health, Australia; School of Translational Medicine, Faculty of Medicine, Nursing and Health Science, Monash University, Melbourne, Australia; Melbourne Sexual Health Centre, Alfred Health, Australia; School of Translational Medicine, Faculty of Medicine, Nursing and Health Science, Monash University, Melbourne, Australia; Centre for Epidemiology and Biostatistics, Melbourne School of Population and Global Health, The University of Melbourne, Melbourne, Australia; The Kirby Institute, University of New South Wales, Sydney, Australia; The Kirby Institute, University of New South Wales, Sydney, Australia; The Kirby Institute, University of New South Wales, Sydney, Australia; Melbourne Sexual Health Centre, Alfred Health, Australia; School of Translational Medicine, Faculty of Medicine, Nursing and Health Science, Monash University, Melbourne, Australia; Melbourne Sexual Health Centre, Alfred Health, Australia; School of Translational Medicine, Faculty of Medicine, Nursing and Health Science, Monash University, Melbourne, Australia; China-Australia Joint Research Center for Infectious Diseases, School of Public Health, Xi’an Jiaotong University Health Science Center, China; Key Laboratory for Disease Prevention and Control and Health Promotion of Shaanxi Province, Xi’an Jiaotong University, China; The Interdisciplinary Center for Mathematics and Life Sciences, School of Mathematics and Statistics, Xi’an Jiaotong University, China; Key Laboratory of Environment and Genes Related to Diseases (Xi’an Jiaotong University), Ministry of Education, China; Phase I Clinical Trial Research Ward, The Second Affiliated Hospital of Xi’an Jiaotong University, Xi'an, China; China-Australia Joint Research Center for Infectious Diseases, School of Public Health, Xi’an Jiaotong University Health Science Center, China; Melbourne Sexual Health Centre, Alfred Health, Australia; School of Translational Medicine, Faculty of Medicine, Nursing and Health Science, Monash University, Melbourne, Australia

**Keywords:** cost-effectiveness, digital anorectal examination, early detection, men who have sex with men, syphilis

## Abstract

**Background:**

Rising syphilis incidence among Australian gay, bisexual, and other men who have sex with men (GBMSM) requires new early detection strategies. Regular self-digital anorectal examination (self-DARE) may facilitate syphilis identification, potentially reducing transmission. We evaluated its population-level impact and cost-effectiveness in controlling syphilis among Australian GBMSM.

**Methods:**

We developed an integrated transmission-dynamic and health-economic model, calibrated with 2012–2022 Australian GBMSM data. Over a 10-year period (2025–2034), we compared the base case with two scenarios: recommending self-DARE to men with higher sexual activity (“only high group”) or to all individuals (“both groups”). We assessed changes in incidence, incremental cost-effectiveness ratios (ICERs), and benefit-cost ratios.

**Results:**

The base case projected 110 501 new infections over 10 years. The “only high group” strategy averted 57 115 infections (51.7%) and was cost saving (negative ICER), with a benefit-cost ratio of 2.5. The “both groups” strategy averted more infections (58 216; 52.7%) but was less economically efficient (benefit-cost ratio: 1.6), though also cost-saving. Sensitivity analyses indicated that improving self-DARE sensitivity enhanced its performance.

**Conclusions:**

Self-DARE could effectively and cost-effectively reduce syphilis among GBMSM, particularly when focused on men with higher sexual activity. Further empirical research is needed to confirm its feasibility and effectiveness.

## Teaser

Our modeling study demonstrated that self-digital anorectal examination could effectively and cost-effectively reduce syphilis infections among Australian gay, bisexual, and other men who have sex with men, particularly when targeted at men with higher sexual activity, offering significant public health benefits.

**(See the Editorial Commentary by Allen on pages e359–61.)**

Syphilis is a curable sexually transmitted infection caused by the bacterium *Treponema pallidum* [1]. In recent decades, syphilis cases have been increasing rapidly in high-income countries, particularly among gay, bisexual, and other men who have sex with men (GBMSM) [2–4]. In Australia, the incidence of infectious syphilis in GBMSM doubled between 2012 and 2022, rising from 2.2 to 4.6 cases per 100 person-years [5]. In response to these trends, the World Health Organization has set a target to reduce syphilis incidence by 90% by 2030 as compared with the 2020 level [6].

Syphilis progresses through distinct stages, each with unique characteristics [7]. Primary syphilis typically manifests as a painless chancre. Among GBMSM, sexual practices influence the anatomic distribution of these lesions, which can appear at sites of inoculation, including the penis, anus, rectum, and oral cavity [8, 9]. Detection of anorectal primary syphilis is particularly challenging, as approximately 90% of cases are asymptomatic [10–12]. Previous research indicates that GBMSM who engage exclusively in receptive anal sex are diagnosed with primary syphilis at one-third the rate of those who engage only in insertive anal sex, likely due to missed primary lesions in the anorectum [13]. Delayed diagnosis of anorectal primary syphilis can lead to secondary syphilis, characterized by systemic infection; yet, missed primary and secondary syphilis can progress to early latent syphilis, which is mostly asymptomatic but has a long infectious period, thereby increasing the potential for further transmission [1]. Consequently, early detection of anorectal primary syphilis, followed by prompt diagnosis and treatment, is crucial in preventing disease progression and reducing infectiousness [14].

Current strategies for controlling syphilis among GBMSM include more frequent sexually transmitted infection screening, testing reminder systems, and opt-out syphilis testing during routine HIV care [15–17]. Despite these interventions, syphilis cases continue to rise, prompting the exploration of innovative control strategies, such as focusing on detecting primary syphilis, particularly occult cases [16].

Self-digital anorectal examination (self-DARE) is an emerging approach wherein individuals are trained to perform regular digital self-examinations to identify anorectal abnormalities. A prospective study demonstrated that self-DARE has moderate sensitivity and high specificity for identifying anorectal abnormalities [18]. In addition, it has been recommended for early anal cancer detection in GBMSM aged >50 years with HIV [19–22]. Since anal cancer and primary syphilis often present as macroscopic palpable lesions within the anorectal area, regular self-DARE may also hold promise for the early detection of anorectal primary syphilis [23–26]. Previous research found self-DARE to be highly acceptable for syphilis detection [23, 24] and associated with high adherence rates among GBMSM [25, 26].

To date, no study has evaluated whether regular practice of self-DARE at a population level could effectively and cost-effectively decrease syphilis incidence among GBMSM. To address this gap, we developed an integrated transmission-dynamic and health-economic model to assess the impact and cost-effectiveness of implementing self-DARE as a public health intervention among GBMSM in Australia. Our findings will provide critical evidence to inform future guidelines and policies regarding the use of self-DARE as a strategy to curb syphilis transmission.

## METHODS

### Data Source

Model parameters, including the population size of GBMSM, sexual activity levels, and natural disease progression rates of syphilis, were obtained from the literature [1, 27–29] and are listed in Supplementary Table 1. Annual rates of testing and infectious syphilis incidence from 2012 to 2022 among Australian GBMSM were derived from published studies based on the ACCESS system (Australian Collaboration for Coordinated Enhanced Sentinel Surveillance) [5, 30]. These annual estimates were used for model calibration to get the point and range estimates of unknown parameters, such as rates of sexual partner change, stage-specific probabilities of syphilis transmission per partnership, the proportion of new infections progressing to anorectal primary syphilis, and stage-specific testing rates.

### Model Construction

**Figure 1.**
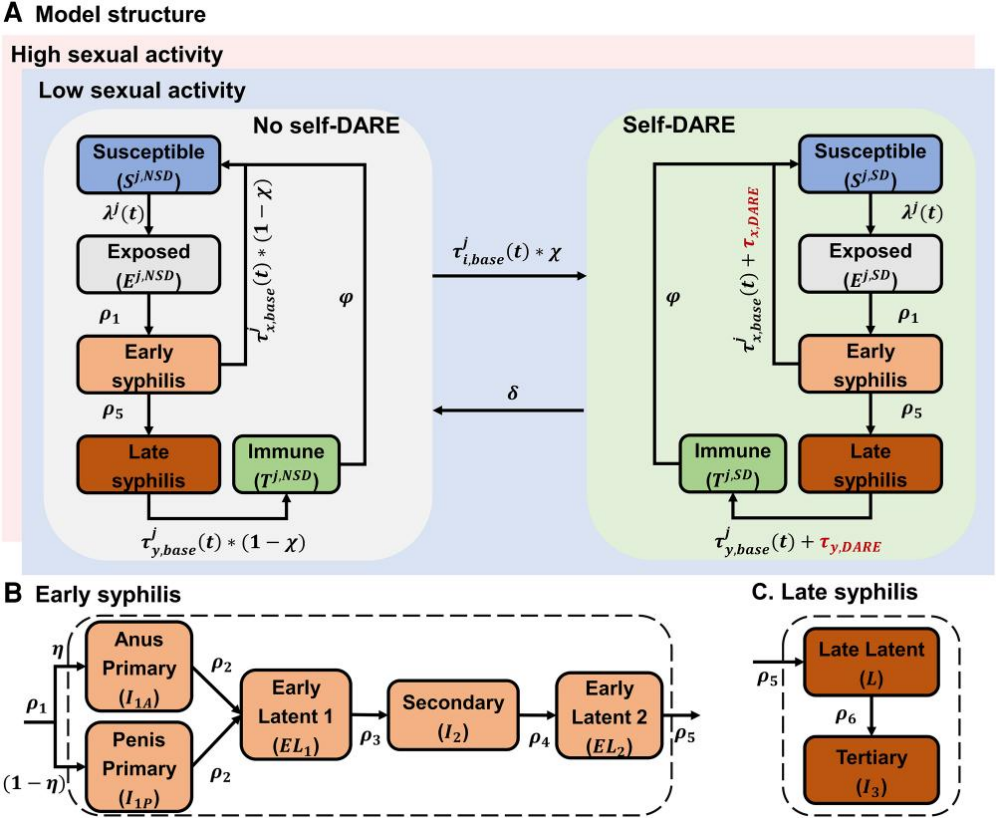
Flowchart of the syphilis transmission model. The model incorporated heterogeneity in sexual behavior, categorizing individuals into groups with low and high sexual activity. In each group, the population was divided into compartments: susceptible (*S*), exposed (*E*), anorectal primary syphilis ($I_{1A}$), penile primary syphilis ($I_{1P}$), first early latent syphilis ($EL_{1}$), secondary syphilis ($I_{2}$), second early latent syphilis ($EL_{2}$), late latent syphilis (*L*), tertiary syphilis ($I_{3}$), and immunity following treatment of late syphilis (*T*). We defined $\lambda^{\;j}(t)(\;j\in \{L,H\})$ as the force of syphilis infection (ie, the probability of the susceptible population becoming infected) in high (*H*) and low (*L*) sexual activity. $\rho_{m}(m\in \{1,2,\ldots ,6\})$ represents the disease progression rates, with the proportion of individuals at the exposed stage progressing to primary syphilis at the anorectum and the penis, denoted as *η* and (1−η), respectively. $\tau_{i,base}^{\;j}(t)(y\in \{1,2,\ldots ,7\})$ indicates the syphilis testing rates among individuals at different infection stages (including those who are susceptible), in which the $\tau_{x,base}^{\;j}(t)(y\in \{1,2,3,4,5\})$ and $\tau_{y,base}^{\;j}(t)(y\in \{6,7\})$ are the testing rates of early syphilis (primary, secondary, and early latent syphilis) and late syphilis (late latent and tertiary syphilis), respectively. After treatment, individuals with early syphilis return to the susceptible state, whereas those with late syphilis gain temporary immunity and gradually revert to susceptibility at a rate φ. When self-DARE is introduced, persons attending syphilis testing can join the self-DARE group (*SD*) at an uptake rate of *χ*. People in the self-DARE group have additional testing rates $\tau_{i,DARE}(t)(y\in \{1,2,\ldots ,7\})$ and return to the no self-DARE group (*NSD*) at a rate of *δ*. Abbreviation: Self-DARE, self-digital anorectal examination.

We developed a deterministic transmission-dynamic compartmental model of syphilis to simulate future epidemic size among GBMSM in Australia under different self-DARE scenarios. The model incorporated heterogeneity in sexual behavior, categorizing individuals into groups with low and high sexual activity, with the latter defined by higher rates of sexual partner change (>10 partners per year) and a greater likelihood of attending sexually transmitted infection testing. In each group, the population was divided into compartments (Figure 1): susceptible (*S*), exposed (*E*), anorectal primary syphilis ($I_{1A}$), penile primary syphilis ($I_{1P}$), first early latent syphilis ($EL_{1}$), secondary syphilis ($I_{2}$), second early latent syphilis ($EL_{2}$), late latent syphilis (*L*), tertiary syphilis ($I_{3}$), and immunity following treatment of late syphilis (*T*). We defined $\lambda^{\;j}(t)(\;j\in \{L,H\})$ as the force of syphilis infection (ie, the probability of the susceptible population becoming infected) in high (H) and low (L) sexual activity. $\rho_{m}(m\in \{1,2,\ldots ,6\})$ represents the disease progression rates, with the proportion of individuals at the exposed stage progressing to primary syphilis at the anorectum and at the penis, denoted as *η* and (1−η), respectively. $\tau_{i,base}^{\;j}(t)(y\in \{1,2,\ldots ,7\})$ represents the syphilis testing rates among individuals at different infection stages (including those who are susceptible), in which $\tau_{x,base}^{\;j}(t)(y\in \{1,2,3,4,5\})$ and $\tau_{y,base}^{\;j}(t)(y\in \{6,7\})$ are the testing rates of early syphilis (primary, secondary, and early latent syphilis) and late syphilis (late latent and tertiary syphilis). After treatment, individuals with early syphilis return to the susceptible state, whereas those with late syphilis gain temporary immunity and gradually revert to susceptibility at a rate φ.

When self-DARE is introduced, persons attending syphilis testing can join the self-DARE group at an uptake rate of *χ*. Those in the self-DARE group have additional testing rates $\tau_{i,DARE}(t)(y\in \{1,2,\ldots ,7\})$ and return to the “no self-DARE” group at a rate of *δ*. Further methodological details are provided in the supplementary material.

### Model Parametrization and Calibration

Prior information from the literature was integrated into the model. Unknown epidemiologic parameters were inferred by calibrating the model to annual testing rates and infectious syphilis incidence. Using the Markov chain Monte Carlo method, we obtained samples from the joint posterior distribution of parameters and selected 1000 parameter sets for subsequent simulations. Prior and posterior distributions of these parameters are listed in Supplementary Table 2. Postcalibration (Supplementary Figure 1), our model replicated observed trends in increasing sexual risk behaviors and screening rates [5, 28]. All procedures and analyses were performed in Python (version 3.11.9). Additional details are presented in the supplementary material.

### Model Simulation of the Regular Self-DARE

We simulated the introduction of regular self-DARE in 2025 and evaluated its impact over a 10-year period (2025–2034). In addition to a base case scenario without self-DARE, we evaluated 2 intervention strategies: (1) the “only high group” strategy, where self-DARE was recommended exclusively for individuals in the high-activity group presenting for syphilis testing; (2) the “both groups” strategy, where self-DARE was recommended for all attending syphilis testing (ie, those in high- and low-activity groups).

Regular self-DARE was modeled as an additional self-screening approach. Participants were assumed to detect abnormal anorectal findings (including primary syphilis at the anorectum), leading to more frequent clinical visits, testing, diagnosis, and treatment. Given previous studies, we assumed a 180-day intervention duration, a screening frequency of once every 30 days, and sensitivity and specificity of 60% and 80%, respectively [18, 23, 26]. Parameter values were informed by a 12-week cohort study at the Melbourne Sexual Health Centre involving 30 GBMSM [26]. The uptake was set at 72%, as suggested by a previous discrete choice experiment [25]. We then predicted new infections, testing volumes, and syphilis diagnoses under the base case and the 2 self-DARE strategies. Additional simulation details are provided in the supplementary material.

### Economic Evaluation

According to the Australian sexually transmitted infection guidelines [31], we concluded the cost composition of syphilis diagnosis and treatment among GBMSM in Australia as follows:

Primary diagnosis: 1 level B general practitioner consultation [32] and 1 single syphilis-specific antibody test
Confirmatory diagnosis: 1 level C general practitioner consultation [33], 1 double supplemental antibody test, and 1 nucleic acid amplification test for *T pallidum*
Treatment of syphilis: 1 single antibody test before treatment, 1 level B general practitioner consultation (3 for late syphilis), and 2 injections of benzathine benzylpenicillin (6 for late syphilis)

We estimated the costs of syphilis testing and syphilis treatment from the Australian Medicare Benefits Schedule Book and Pharmaceutical Benefits Scheme [34, 35].

For the cost of the self-DARE, we considered the cost of engaging GBMSM to participate in the intervention (link to care), which was set at A$50 based on the empirical study [26], and the additional costs of syphilis clinical review, diagnosis, and treatment due to self-DARE. For the health benefit, we considered the overall quality-adjusted life-year (QALY) loss of 0.06 caused by each new syphilis infection among GBMSM according to a previous survey [36].

We calculated the incremental costs and incremental QALYs for self-DARE strategies as compared with no self-DARE (base case) over the next 10 years. We defined the incremental cost per QALY gained as the incremental cost-effectiveness ratio (ICER), which would be compared with the cost-effectiveness threshold of A$50 000. We also calculated the cost per infection averted and the benefit-cost ratios. This cost-effective analysis, conducted from the perspective of the health care system, values all monetary costs and benefits in 2024 Australian dollars and discounts them with QALYs at a 3% rate.

### Sensitivity Analysis

We performed sensitivity analyses for the only high group and both groups strategies, examining variations in uptake (35%–98%) [25], screening frequency (every 7, 30, or 90 days), and self-DARE sensitivity and specificity (10%–100%). We also conducted 1-way sensitivity analyses on additional parameters to identify key drivers of health-economic outcomes.

## RESULTS

### Epidemic Impact of Self-DARE on Syphilis

**Figure 2.**
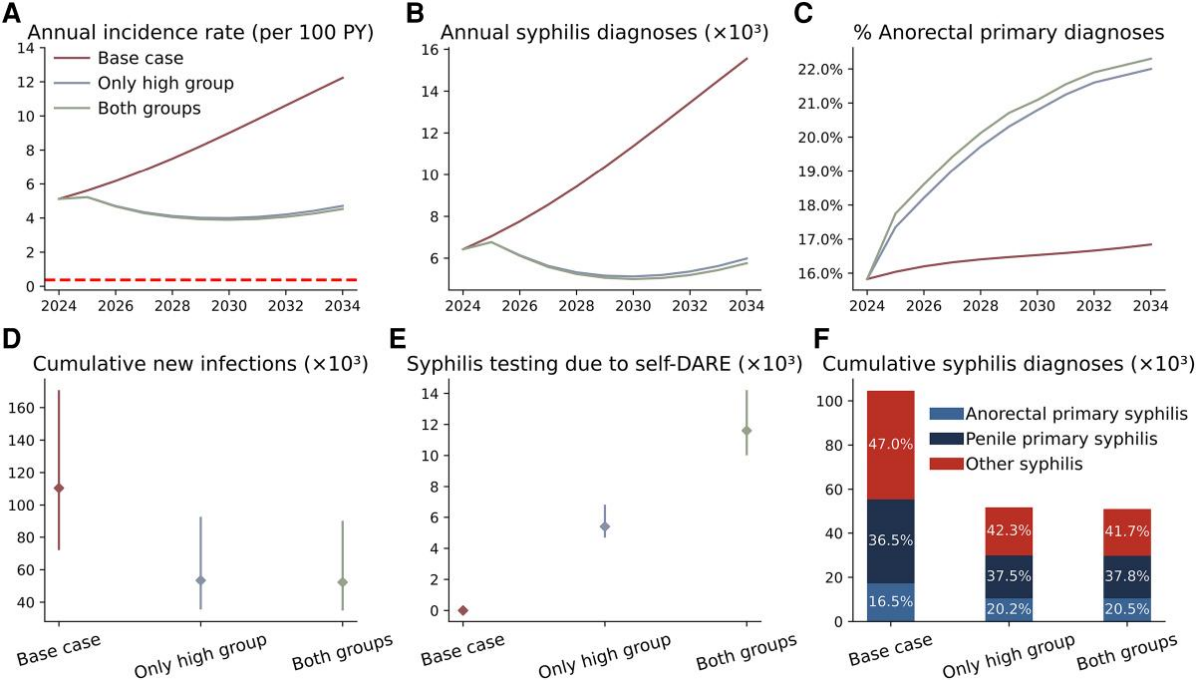
Population-level impact of self-DARE on syphilis incidence, diagnoses, and testing. *A*, Annual incidence of syphilis per 100 person-years under various scenarios, with a red dashed line indicating an incidence of 0.37 per 100 person-years, which represents the World Health Organization's 2030 target of a 90% reduction from the projected 2020 incidence of 3.7 per 100 person-years. *B*, Annual number of syphilis diagnoses across different scenarios. *C*, Proportion of anorectal primary syphilis among annual diagnoses. *D*, Cumulative new infections from 2025 to 2034, with points showing medians and bars 95% credible intervals. *E*, Cumulative syphilis tests prompted by self-DARE. *F*, Cumulative syphilis diagnoses from 2025 to 2034, stratified by syphilis stage.

We identified substantial reductions in new syphilis infections under the 2 self-DARE strategies as compared with the base case (Figure 2). In the base case, we projected 110 501 (95% CI, 72 718–169 885) new syphilis infections between 2025 and 2034. By implementing self-DARE with the only high group strategy, the cumulative number of infections would be reduced by 57 115 (35 254–72 073), corresponding to a 51.7% reduction from the base case. With the both groups strategy, infections would be reduced by 58 216 (38 105–75 831), corresponding to a 52.7% reduction.

According to the World Health Organization’s (WHO's) target of reducing syphilis incidence by 90% by 2030 as compared with the 2020 level, we estimated that Australian GBMSM would need to reach an incidence of 0.32 per 100 person-years by 2030. Implementing self-DARE with the only high group and both groups strategies would lower incidence to 4.1 and 3.9 per 100 person-years, respectively, neither of which meets the WHO target.

In the base case, we projected 109 741 (95% CI, 70 554–158 931) syphilis diagnoses between 2025 and 2034. By implementing self-DARE with the only high group strategy, cumulative diagnoses would decrease by 55 670 (from 109 741 to 54 071), corresponding to a 50.7% reduction. Under the both groups strategy, cumulative diagnoses would decrease by 56 837 (from 109 741 to 52 904), corresponding to a 51.8% reduction.

In the base case, anorectal primary syphilis, penile primary syphilis, and other syphilis accounted for 16.5%, 36.5%, and 47.0% of total diagnoses, respectively. With the only high group strategy, these proportions would shift to 20.2%, 37.5%, and 42.3%. Under the both groups strategy, the proportions would be 20.5%, 37.8%, and 41.7%. These changes reflect a relative increase in the proportion of anorectal primary syphilis detected, as more cases would be identified earlier through self-DARE.

By implementing the intervention with the only high group and both groups strategies, there would be 5613 (4705–7594) and 11 690 (10 031–14 152) syphilis tests prompted by abnormalities detected through self-DARE, respectively.

### Cost-effectiveness of Self-DARE

**Figure 3.**
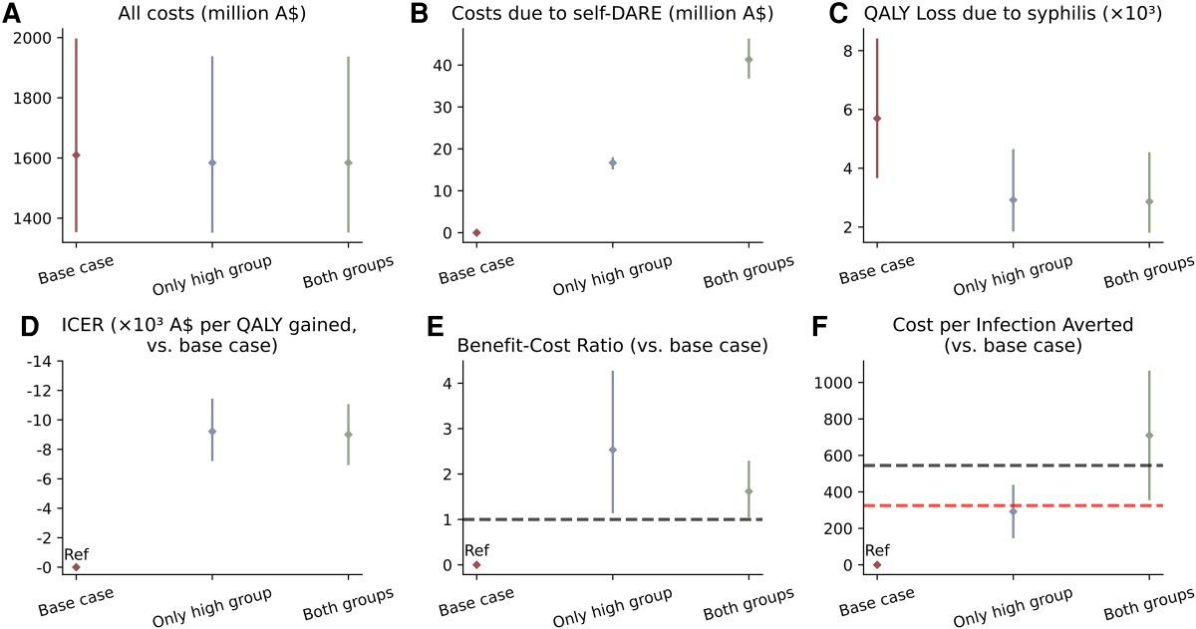
Health-economic outcomes of self-DARE in controlling the syphilis epidemic among GBMSM from 2025 to 2034. *A*, The cumulative costs of screening, syphilis management, and self-DARE. *B*, The cumulative costs due to self-DARE. *C*, The cumulative loss of quality-adjusted life-years due to syphilis. *D*, Incremental cost-effectiveness ratios as compared with the base case (no self-DARE). *E*, Benefit-cost ratios as compared with the base case (no self-DARE), with a black dashed line indicating a benefit-cost ratio of 1. *F*, Cost per infection averted as compared with the base case (no self-DARE), with red and black dashed lines representing the costs of diagnosing and treating 1 early syphilis case (A$325) and 1 late syphilis case (A$545), respectively. All outcomes are discounted, with points representing medians and bars 95% credible intervals. Abbreviations: GBMSM, gay, bisexual, and other men who have sex with men; ICER, incremental cost-effectiveness ratio; QALY, quality-adjusted life-year; self-DARE, self-digital anorectal examination.

We calculated the costs and QALY loss between 2025 and 2034 for the base case and for the self-DARE scenarios (Figure 3). The total costs of the base case, only high group, and both groups scenarios were, in Australian dollars, 1609.2 million (95% CI, 1354.5–1997.4), 1583.0 million (1351.3–1935.7), and 1585.0 million (1352.6–1937.2), respectively. The costs attributable to self-DARE in the only high group and both groups scenarios were 16.7 million (15.1–18.0) and 41.3 million (36.8–46.3). We estimated QALY losses of 5695.2 (3666.1–8414.0), 2922.6 (1845.3–4652.6), and 2864.5 (1807.1–4543.2) in the base case, only high group, and both groups scenarios.

As compared with the base case, implementing self-DARE with the only high group and both groups strategies would yield, in Australian dollars, ICERs of –9218.1 (95% CI, −11 453.2 to −7214.5) and –9007.2 (−11 075.9 to −6931.4), respectively, both below zero, indicating that these strategies are cost saving. In addition, these 2 strategies would result in benefit-cost ratios of 2.5 (1.1–4.3) and 1.6 (1.0–2.3), both >1. They could avert 1 new syphilis infection at costs of 292.3 (146.1–438.4) and 709.8 (354.9–1064.6), with the former being below the cost of diagnosing and treating 1 early syphilis case (325).

### Sensitivity Analysis

**Figure 4.**
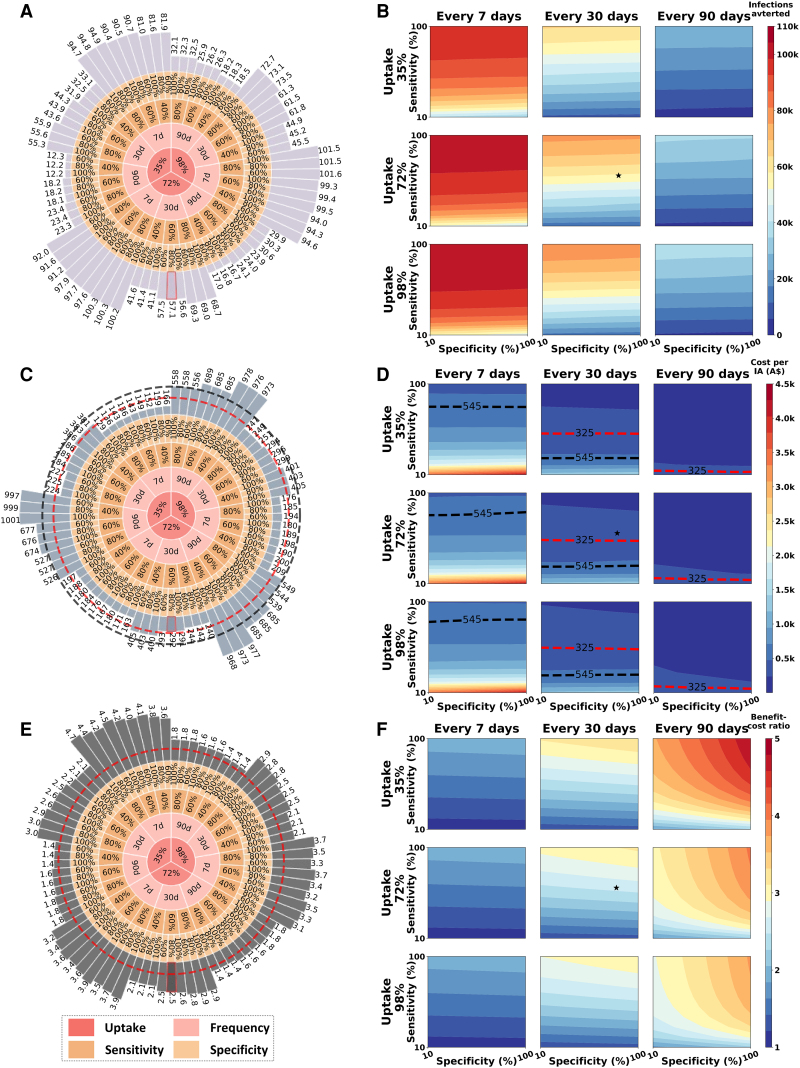
Sensitivity analysis of the epidemiologic and health-economic outcomes in scenarios providing self-DARE only for high-activity GBMSM. *A***–***B*, The number of cumulative infections averted between 2025 and 2034. *C–D*, The cost per infection averted by implementing self-DARE, with dashed lines representing the costs of diagnosing and treating 1 early syphilis case (A$325) and 1 late syphilis case (A$545), respectively. *E–F*, The benefit-cost ratios, with dashed line indicating a benefit-cost ratio of 1. The bars with red frames in panels *A*, *C*, and *E* and the pentagrams in panels *B*, *D*, and *F* represent the outcomes in “only high group” scenario with base estimates of the features. Abbreviations: GBMSM, gay, bisexual, and other men who have sex with men; self-DARE, self-digital anorectal examination.

Our findings were relatively robust to parameter uncertainty (Figure 4, Supplementary Figures 2–5). In the sensitivity analysis of the only high group scenario, varying uptake (35%, 72%, 98%), frequency (once every 7, 30, or 90 days), sensitivity (10%–100%), and specificity (10%–100%), we found that ICERs remained below zero (Supplementary Figure 2). Across these variations, the only high group strategy could avert 2900 to 102 871 new infections (Figure 4*A*–*B*), leading to costs per infection averted of 126.3 to 4219.8 Australian dollars (Figure 4*C*–*D*) and benefit-cost ratios of 1.1 to 4.8 (Figure 4*E*–*F*). Under varying conditions, the only high group strategy could avert 1 syphilis infection for <325 Australian dollars (the cost of diagnosing and treating early syphilis) or <545 (the cost of diagnosing and treating late syphilis) in 46.7% and 68.7% of simulations, respectively (Figure 4*D*).

Increasing the frequency of self-DARE averted more infections but also raised the cost per infection averted and lowered the benefit-cost ratio in the only high group scenario. For example, increasing the frequency from once every 90 days (right column of Figure 4*B*, *D*, *F*) to once every 7 days (left column of Figure 4*B*, *D*, *F*) increased the number of infections averted from 2900–38 901 to 33 450–102 933. Simultaneously, the cost per infection averted rose from 126.3–400.2 Australian dollars to 435.6–4219.8, and the benefit-cost ratio decreased from 1.8–4.8 to 1.1–2.0. Increasing the uptake averted more infections but had less impact on cost per infection averted and the benefit-cost ratio.

Higher sensitivity of self-DARE led to more infections averted, a lower cost per infection averted, and higher benefit-cost ratios in the only high group scenario. Increasing sensitivity from 10% (bottom of each subpanel in Figure 4*B*, *D*, *F*) to 100% (top of each subpanel) raised the number of infections averted from 2900–55 631 to 28 099–102,933, decreased the cost per infection averted from 364.2–4219.8 Australian dollars to 126.3–469.9, and increased the benefit-cost ratio from 1.1–2.2 to 1.9–4.8. In contrast, changes in specificity did not markedly alter the number or cost per infection averted, but higher specificity increased the benefit-cost ratio, particularly at a frequency of once every 90 days. By raising specificity from 10% (left of each subpanel in Figure 4*F*) to 100% (right of each subpanel), the benefit-cost ratio increased from 1.1–3.7 to 1.1–4.8.

One-way sensitivity analyses identified the cost of engaging GBMSM to participate in the intervention (link to care) as a key factor affecting cost per infection averted and benefit-cost ratio (Supplementary Figure 3). With this cost ranging from 0 to 100 Australian dollars, the costs per infection averted would increase from 11.1 to 769.3, and the benefit-cost ratios would decrease from 8.2 to 1.8.

In the both groups scenario, varying uptake, frequency, sensitivity, and specificity also led to a higher number of infections averted, a higher cost per infection averted, and a lower benefit-cost ratio when compared with the only high group scenario. Similar patterns in how frequency, uptake, sensitivity, and specificity influenced these outcomes in the only high group scenario were observed in the both groups scenario.

## DISCUSSION

In this modeling study, we evaluated the potential impact and cost-effectiveness of implementing self-DARE among Australian GBMSM under 2 strategies: recommending regular self-DARE only to men with high sexual activity (only high group) and recommending regular self-DARE to all men attending syphilis testing (both groups). Our findings show that both strategies can substantially reduce new syphilis infections and diagnoses over a 10-year period (2025–2034) while remaining cost saving as compared with the base case (no self-DARE).

In terms of epidemic impact, both self-DARE strategies resulted in an approximately 50% reduction in cumulative new syphilis infections and diagnoses, relative to a base case without self-DARE. Although these reductions are considerable, neither strategy reached the WHO's ambitious 2030 target of a 90% reduction in incidence as compared with the 2020 level. Notably, while the strategies provided similar overall reductions in new infections, focusing self-DARE on the high sexual activity group was more economically efficient. Specifically, the only high group strategy had a lower cost per infection averted and a higher benefit-cost ratio as compared with recommending self-DARE more broadly. These findings suggest that a more targeted application of self-DARE could yield greater economic value while still significantly curbing transmission.

By prompting clinical follow-up visits in response to perceived anorectal abnormalities, self-DARE increased the proportion of primary syphilis (anorectal or penile) detected among all syphilis diagnoses. When an individual undertakes a self-DARE and finds an abnormality, regardless of whether it is a true positive (primary syphilis) or false positive (eg, anal wart, anal fissure, or herpes), the individual would attend the clinic for clinical review and syphilis testing. This would increase the likelihood of diagnosing anorectal primary syphilis through true positives of self-DARE and could lead to a slight increase in the diagnosis of other syphilis cases from testing when false positives of self-DARE occur. Thus, even imperfect specificity can indirectly support earlier case detection and reduce the spread of infection.

The only high group and both groups strategies were cost saving when compared with the base case, with negative ICERs and benefit-cost ratios exceeding 1. The only high group strategy was particularly cost-effective, with cost per infection averted (A$292.3) below that of diagnosing and treating an early syphilis case. Although extending self-DARE to all attendees averted slightly more infections, it came at a higher cost per infection averted (A$709.8), diminishing its relative economic advantage.

Sensitivity analyses emphasized the importance of sensitivity and specificity of self-DARE. Improving sensitivity notably increased the number of infections averted and enhanced cost-effectiveness. In contrast, while higher specificity did not alter the number or cost per infection averted, it improved the benefit-cost ratio, particularly at lower screening frequencies, potentially because greater specificity reduces unnecessary clinical evaluations and testing costs for individuals without syphilis [37]. We selected a sensitivity of 60% and a specificity of 80% as our base estimates, drawn from a population study assessing the accuracy of self-DARE in detecting anorectal abnormalities [18], and these values may be further optimized through more comprehensive participant education and training [18, 23]. Future research should focus on strategies to improve self-DARE performance.

Frequency and uptake could shape the impact and cost-effectiveness of self-DARE. Increasing the frequency of self-DARE averted more infections but raised the cost per infection averted and reduced the benefit-cost ratio, whereas higher uptake led to greater reductions in infections with less impact on cost metrics. Monthly self-DARE was based on GBMSM preferences from a discrete choice experiment [25], but weekly self-DARE may be feasible given the high adherence observed in a small study of 30 GBMSM [26]. While weekly self-DARE could double the number of infections averted, it would quadruple the cost per infection averted and reduce the benefit-cost ratio by one-third. Ensuring greater convenience, offering timely clinical review, and recommending self-DARE to groups more likely to have anorectal primary syphilis (eg, receptive-only GBMSM) could improve uptake [25]. Additionally, we identified the cost of engaging GBMSM to participate in self-DARE as a crucial determinant of cost-effectiveness. Integrating self-DARE into routine health education could lower these engagement costs and enhance the intervention's value.

This study has several limitations. First, we modeled only primary syphilis in the penis or anorectum, excluding oral lesions that may play an important role in transmission. As a result, we might overestimate the impact of self-DARE if oral lesions substantially contribute to the epidemic. Second, our sensitivity and specificity estimates were based on a single study assessing the accuracy of self-DARE for detecting anorectal abnormalities not specific to syphilis—currently the only data available. Although we conducted extensive sensitivity analyses to explore the effects of varying these parameters, more targeted data are needed for improved precision. Third, we did not stratify uptake by individual characteristics (eg, risk of syphilis) that may influence the likelihood of anorectal primary syphilis detection, potentially leading to imprecise estimates of the intervention's overall effect. Fourth, due to limited data, we did not consider secondary lesions in the anorectal area, which may lead to underestimation of the impacts and cost-effectiveness of self-DARE, highlighting the need for further research to refine the model. Finally, we did not consider the potential benefits of reducing syphilis among GBMSM on women, including the prevention of congenital syphilis—a severe and costly outcome [38]. Therefore, our results may underestimate the full public health and economic value of self-DARE.

## CONCLUSION

Self-DARE has the potential to effectively and cost-effectively reduce syphilis infections among Australian GBMSM. Targeting individuals with higher sexual activity achieves similar reductions in syphilis infections as compared with implementing self-DARE for all GBMSM, while offering notable economic advantages. Ensuring high sensitivity, specificity, and uptake of self-DARE is crucial to maximizing its impact and cost-effectiveness. Further empirical research is necessary to validate its real-world effectiveness and explore the feasibility of achieving the required levels of uptake and frequency at the population level.

## Supplementary Material

jiaf310_Supplementary_Data

## Supplementary Data

Supplementary materials are available at *The Journal of Infectious Diseases* online (http://jid.oxfordjournals.org/). Supplementary materials consist of data provided by the author that are published to benefit the reader. The posted materials are not copyedited. The contents of all supplementary data are the sole responsibility of the authors. Questions or messages regarding errors should be addressed to the author.

## Notes

***Acknowledgments.*** The authors gratefully acknowledge Professor Mark A. Stoové from the Burnet Institute for support in collecting the data.

***Author contributions.*** C. K. F. conceived the concept of self-DARE for the control of syphilis in GBMSM. L. Z., C. K. F., and M. S. conceived the study. L. Z. and M. S. designed the study. H. L. collected and analyzed the data. H. L. carried out the analysis and performed numeric simulations. H. L. wrote the first draft of the manuscript. L. Z., C. K. F., and M. S. critically revised the manuscript. All authors contributed to writing the manuscript and agreed with the results and conclusions.

***Supplementary material.*** The supplementary material encompasses the surveillance data utilized for model calibration, precise values of the parameters with their sources, and additional results.

***Financial support.*** This work was supported by the Ministry of Science and Technology of the People's Republic of China (2022YFC2304900 and 2022YFC2304905 to L. Z.); the National Key R&D Program of China (2022YFC2505100 and 2022YFC2505103 to L. Z.); the National Natural Science Foundation of China (12171387 to M. S.); the National Key R&D Program of China (2022YFC2505100 to M. S.); an Australian NHMRC Leadership Investigator Grant (GNT1172900 to C. K. F.); an NHMRC Practitioner Fellowship (GNT1154828 to B. D.); and an NHMRC Emerging Leadership Investigator Grant (GNT1172873 to E. P. F. C.).

## References

[1] French P. Syphilis. BMJ 2007; 334:143–7. 17235095 10.1136/bmj.39085.518148.BE PMC1779891

[2] Rowley J, Vander Hoorn S, Korenromp E, et al Chlamydia, gonorrhoea, trichomoniasis and syphilis: global prevalence and incidence estimates, 2016. Bull World Health Organ 2019; 97:548–62P. 31384073 10.2471/BLT.18.228486 PMC6653813

[3] Williamson DA, Chen MY. Emerging and reemerging sexually transmitted infections. N Engl J Med 2020; 382:2023–32. 32433838 10.1056/NEJMra1907194

[4] Chow EPF, Grulich AE, Fairley CK. Epidemiology and prevention of sexually transmitted infections in men who have sex with men at risk of HIV. Lancet HIV 2019; 6:e396–405. 31006612 10.1016/S2352-3018(19)30043-8

[5] Traeger MW, Guy R, Taunton C, et al Syphilis testing, incidence, and reinfection among gay and bisexual men in Australia over a decade spanning HIV PrEP implementation: an analysis of surveillance data from 2012 to 2022. Lancet Reg Health West Pac 2024; 51:101175. 39263009 10.1016/j.lanwpc.2024.101175 PMC11387360

[6] World Health Organization. Regional action plans for ending AIDS and the epidemics of viral hepatitis and sexually transmitted infections 2022–2030. Available at: https://www.who.int/europe/publications/i/item/9789289058957. Accessed 6 November 2024.

[7] Peeling RW, Mabey D, Kamb ML, Chen X-S, Radolf JD, Benzaken AS. Syphilis. Nat Rev Dis Primers 2017; 3:17073. 29022569 10.1038/nrdp.2017.73 PMC5809176

[8] Morshed MG. Current trend on syphilis diagnosis: issues and challenges. Adv Exp Med Biol 2014; 808:51–64. 24595610 10.1007/978-81-322-1774-9_5

[9] Towns JM, Chow EPF, Wigan R, et al Anal and oral detection of *Treponema pallidum* in men who have sex with men with early syphilis infection. Sex Transm Infect 2022; 98:570–4. 35618414 10.1136/sextrans-2021-055370

[10] Fuchs W, Kreuter A, Hellmich M, et al Asymptomatic anal sexually transmitted infections in HIV-positive men attending anal cancer screening. Br J Dermatol 2016; 174:831–8. 26577338 10.1111/bjd.14288

[11] Aung ET, Chen MY, Fairley CK, et al Spatial and temporal epidemiology of infectious syphilis in Victoria, Australia, 2015–2018. Sex Transm Dis 2021; 48:e178–82. 33859143 10.1097/OLQ.0000000000001438

[12] Gunn RA. Expedited intervention services for possible occult primary syphilis among MSM with asymptomatic incident syphilis. Sex Transm Dis 2009; 36:594–5. 19661841 10.1097/OLQ.0b013e3181b36276

[13] Cornelisse VJ, Chow EPF, Latimer RL, et al Getting to the bottom of it: sexual positioning and stage of syphilis at diagnosis, and implications for syphilis screening. Clin Infect Dis 2020; 71:318–22. 31420649 10.1093/cid/ciz802

[14] Chow EPF, Callander D, Fairley CK, et al Increased syphilis testing of men who have sex with men: greater detection of asymptomatic early syphilis and relative reduction in secondary syphilis. Clin Infect Dis 2017; 65:389–95. 28419198 10.1093/cid/cix326

[15] Golden MR, Dombrowski JC. Syphilis control in the postelimination era: implications of a new syphilis control initiative for sexually transmitted disease/human immunodeficiency virus programs. Sex Transm Dis 2018; 45(9S):S86–92. 30102682 10.1097/OLQ.0000000000000775 PMC6002884

[16] Gunn RA, Klausner JD. Enhancing the control of syphilis among men who have sex with men by focusing on acute infectious primary syphilis and core transmission groups. Sex Transm Dis 2019; 46:629–36. 31356529 10.1097/OLQ.0000000000001039 PMC6887624

[17] Peel J, Chow EPF, Denham I, et al Clinical presentation of incident syphilis among men who have sex with men taking HIV pre-exposure prophylaxis in Melbourne, Australia. Clin Infect Dis 2021; 73:e934–7. 33522575 10.1093/cid/ciab052

[18] Nyitray AG, McAuliffe TL, Liebert C, et al The accuracy of anal self- and companion exams among sexual minority men and transgender women: a prospective analysis. Lancet Reg Health Am 2024; 31:100704. 38440068 10.1016/j.lana.2024.100704 PMC10910307

[19] Nyitray AG, D'Souza G, Stier EA, Clifford G, Chiao EY. The utility of digital anal rectal examinations in a public health screening program for anal cancer. J Low Genit Tract Dis 2020; 24:192–6. 31972661 10.1097/LGT.0000000000000508 PMC7147422

[20] Ong JJ, Chen M, Grulich AE, Fairley CK. Regional and national guideline recommendations for digital ano-rectal examination as a means for anal cancer screening in HIV positive men who have sex with men: a systematic review. BMC Cancer 2014; 14:557. 25081485 10.1186/1471-2407-14-557 PMC4137084

[21] Ong JJ, Fairley CK, Carroll S, et al Cost-effectiveness of screening for anal cancer using regular digital ano-rectal examinations in men who have sex with men living with HIV. J Int AIDS Soc 2016; 19:20514. 26942721 10.7448/IAS.19.1.20514 PMC4778406

[22] Ong J, Chen M, Temple-Smith M, et al The inside story: physicians’ views on digital ano-rectal examination for anal cancer screening of HIV positive men who have sex with men. J Med Screen 2013; 20:188–91. 24307004 10.1177/0969141313515463

[23] Aung ET, Fairley CK, Ong JJ, et al Exploring the attitudes of men who have sex with men on anal self-examination for early detection of primary anorectal syphilis: a qualitative study. BMC Infect Dis 2021; 21:982. 34544383 10.1186/s12879-021-06686-4 PMC8453991

[24] Aung ET, Fairley CK, Ong JJ, et al A cross-sectional survey on attitudes of men who have sex with men towards anal self-examination for detection of anal syphilis. Sci Rep 2022; 12:8962. 35624185 10.1038/s41598-022-12881-3 PMC9142515

[25] Aung ET, Chow EPF, Fairley CK, et al Preferences of men who have sex with men for performing anal self-examination for the detection of anal syphilis in Australia: a discrete choice experiment. Lancet Reg Health West Pac 2022; 21:100401. 35243457 10.1016/j.lanwpc.2022.100401 PMC8873922

[26] Aung ET, Fairley CK, Ong JJ, et al Adherence to weekly anal self-examination among men who have sex with men for detection of anal syphilis. Front Med 2022; 9:941041. 10.3389/fmed.2022.941041 PMC9376231 35979212

[27] Callander D, Mooney-Somers J, Keen P, et al Australian “gayborhoods” and “lesborhoods”: a new method for estimating the number and prevalence of adult gay men and lesbian women living in each Australian postcode. Int J Geogr Inf Sci 2020; 34:2160–76.

[28] UNSW. Annual report of trends in behaviour 2024. Available at: https://unsworks.unsw.edu.au/bitstreams/a882159e-b8ef-4b0b-9d63-ba0a2b579f8b/download. Accessed 28 August 2024.

[29] Hui BB, Ward JS, Guy R, Law MG, Gray RT, Regan DG. Impact of testing strategies to combat a major syphilis outbreak among Australian Aboriginal and Torres Strait Islander peoples: a mathematical modeling study. Open Forum Infect Di 2022; 9:ofac119. 10.1093/ofid/ofac119 PMC9035217 35474757

[30] Callander D, Moreira C, El-Hayek C, et al Monitoring the control of sexually transmissible infections and blood-borne viruses: protocol for the Australian Collaboration for Coordinated Enhanced Sentinel Surveillance (ACCESS). JMIR Res Protoc 2018; 7:e11028. 30459142 10.2196/11028 PMC6280029

[31] STI Guidelines Australia. Syphilis. Available at: https://sti.guidelines.org.au/sexually-transmissible-infections/syphilis/. Accessed 13 February 2023.

[32] Medicare Benefits Schedule. Item 23. Available at: https://www9.health.gov.au/mbs/fullDisplay.cfm?type=item&q=23. Accessed 14 July 2024.

[33] Medicare Benefits Schedule. Item 36. Available at: https://www9.health.gov.au/mbs/fullDisplay.cfm?type=item&q=36. Accessed 14 July 2024.

[34] Australian Government, Department of Health and Aged Care. MBS Online. Available at: https://www.mbsonline.gov.au/internet/mbsonline/publishing.nsf/Content/Downloads-240301. Accessed 6 March 2024.

[35] Australian Government, Department of Health and Aged Care. The Pharmaceutical Benefits Scheme. Available at: https://www.pbs.gov.au/browse/downloads. Accessed 6 March 2024.

[36] Lee K, You S, Li Y, et al Estimation of the lifetime quality-adjusted life years (QALYs) lost due to syphilis acquired in the United States in 2018. Clin Infect Dis 2023; 76:e810–9. 35684943 10.1093/cid/ciac427 PMC9907519

[37] Kin C. Anal conditions: STDs. In: Steele SR, Maykel JA, Wexner SD, eds. Clinical decision making in colorectal surgery. Cham: Springer International Publishing; 2020:183–7.

[38] Cunningham SD, Olthoff G, Burnett P, Rompalo AM, Ellen JM. Evidence of heterosexual bridging among syphilis-positive men who have sex with men. Sex Transm Infect 2006; 82:444–5. 17151030 10.1136/sti.2005.019513 PMC2563879

