# Correction to: Impact and Cost-effectiveness of Regular Self-digital Anorectal Examination on Syphilis Among Gay, Bisexual, and Other Men Who Have Sex With Men: A Mathematical Modeling Study

**DOI:** 10.1093/infdis/jiaf465

**Published:** 2025-09-10

This is a correction to: Lai H, Fairley CK, Li R et al., Impact and Cost-effectiveness of Regular Self-digital Anorectal Examination on Syphilis Among Gay, Bisexual, and Other Men Who Have Sex With Men: A Mathematical Modeling Study, *The Journal of Infectious Diseases*, jiaf310, https://doi.org/10.1093/infdis/jiaf310.

In the original publication of this article, the author Mingwang Shen was erroneously listed with affiliations 1, 8, 9, and 10, and should have been listed with affiliations 2, 8, 9, and 10. This has been updated in the version of the article currently available online.

