# Supplementary material for: Impact and Cost-effectiveness of Regular Self-digital Anorectal Examination on Syphilis Among Gay, Bisexual, and Other Men Who Have Sex With Men: A Mathematical Modeling Study
Source: J Infect Dis. 2025 Jul 15;232(3):e393–402. doi: 10.1093/infdis/jiaf310 (PMC12455306; doi:10.1093/infdis/jiaf310)
Supplement: jiaf310_Supplementary_Data [file jiaf310_Supplementary_Data.docx]

**Impact and cost-effectiveness of regular self-digital anorectal examination on syphilis among gay, bisexual, and other men who have sex with men: a mathematical modelling study**

**Supplemental materials:**

1. **Model construction, calibration, and outcome calculation**

**1.1 Model construction**

We developed a deterministic transmission-dynamic compartmental model of syphilis to simulate future epidemic size among GBMSM in Australia under different self-DARE scenarios. The model incorporated heterogeneity in sexual behavior, categorizing individuals into groups with low and high sexual activity, with the latter group defined by higher rates of sexual partner change (more than ten partners per year) and greater likelihood of attending for STI testing. In each group, the population was divided into compartments (**Figure 1**): susceptible ($S$), exposed ($E$), anorectal primary syphilis ($I_{1A}$), penile primary syphilis ($I_{1P}$), first early latent syphilis ($EL_{1}$), secondary syphilis ($I_{2}$), second early latent syphilis ($EL_{2}$), late latent syphilis ($L$), tertiary syphilis ($I_{3}$), and immunity following treatment of late syphilis ($T$). We defined represents the force of syphilis infection (i.e., the probability of the susceptible population becoming infected) in high (H) and low (L) sexual activity. represent the disease progression rates, with the proportion of individuals at exposed stage progressing to primary syphilis at the anorectum and at the penis being denoted as $\eta$ and $\left(1-\eta\right)$, respectively. are the syphilis testing rates among individuals at different infection stages (including the susceptible individuals), in which the and are the testing rates of early syphilis (primary, secondary, and early latent syphilis) and late syphilis (late latent and tertiary syphilis), respectively. After treatment, individuals with early syphilis return to the susceptible state, whereas individuals with late syphilis gain temporary immunity and gradually revert to susceptibility at a rate .

When self-DARE is introduced, individuals attending syphilis testing can join the self-DARE group ($SD$) at an uptake rate of $\chi$. Individuals in the self-DARE group have additional testing rates and return to the no self-DARE group ($NSD$) at a rate of $\delta$.

The model is shown as follows:

where the $\lambda^{j}(t)$ represents the the force of syphilis infection (i.e., the probability of susceptible population being infected) in sexual activity group $j$. As we considered that the first early latent syphilis and late syphilis are non-infectious,^1,2^ so the $\lambda^{j}(t)$ could be calculated as follows:

$$\begin{matrix} \lambda^{L}(t)=\mu_{L}(\zeta\frac{C_{L}}{N_{L}}+(1-\zeta)(\pi_{L}\frac{C_{L}}{N_{L}}+\pi_{H}\frac{C_{H}}{N_{H}})) \\ \lambda^{H}(t)=\mu_{H}(\zeta\frac{C_{H}}{N_{H}}+(1-\zeta)(\pi_{L}\frac{C_{L}}{N_{L}}+\pi_{H}\frac{C_{H}}{N_{H}})) \end{matrix}$$

where the $N_{L}$ and $N_{H}$ are the total number of individuals in low activity group (group L) and high activity group (group H), $\mu_{L}$ and $\mu_{H}$ are t are the annual rates of partner change in group L and group H. $\zeta$is the level of assortativity in sexual mixing between groups, where $\zeta=0$ denotes proportionate mixing and $\zeta=1$ denotes fully assortative contact.^3^ $C_{L}$ and $C_{H}$ are total number of individuals could effectively transmit the infections in groups:

$$\begin{matrix} C_{L}=\beta_{1}(t)(I_{1A}^{L,NSD}+I_{1A}^{L,SD})+\beta_{2}(t)(I_{1P}^{L,NSD}+I_{1P}^{L,SD})+\beta_{3}(t)(E_{L1}^{L,NSD}+E_{L1}^{L,SD}+E_{L2}^{L,NSD}+E_{L2}^{L,SD})+\beta_{4}(t)(I_{2}^{L,NSD}+I_{2}^{L,SD}) \\ C_{H}=\beta_{1}(t)(I_{1A}^{H,NSD}+I_{1A}^{H,SD})+\beta_{2}(t)(I_{1P}^{H,NSD}+I_{1P}^{L,SD})+\beta_{3}(t)(E_{L1}^{H,NSD}+E_{L1}^{H,SD}+E_{L2}^{H,NSD}+E_{L2}^{H,SD})+\beta_{4}(t)(I_{2}^{H,NSD}+I_{2}^{H,SD}) \end{matrix}$$

where the $\beta_{i}(t)(i\in[1,2,3,4])$ represent the per-partner transmission rates of syphilis at different stages, among which the two stages of early latent syphilis are assumed to have similar transmission rate. They were simulated as increasing with time to simulate the increase in transmission risk behaviour:^3^

$$\beta_{i}(t)=\beta_{i}(t_{0})(1+\theta_{\beta}(t-t_{0}))$$

In addition, $\pi_{L}$ and $\pi_{H}$ are the proportions of all partnerships in the population that involve a member of group L and H:

$$\begin{matrix} \pi_{L}=\frac{\mu_{L}N_{L}}{\mu_{L}N_{L}+\mu_{H}N_{H}} \\ \pi_{H}=\frac{\mu_{H}N_{H}}{\mu_{L}N_{L}+\mu_{H}N_{H}} \end{matrix}$$

Finally, the $\tau_{i}^{j,NSD}(t)(i\in\{1,2,...,6\},j\in\{L,H\})$ represent the testing rates of different syphilis compartments not taking self-DARE, among which we assumed that: (1) individuals at susceptible status, immunity status (treated late syphilis) have the same testing rate, (2) exposed individuals would be testing with the same rate as the susceptible individuals, but would not be detected, (3) individuals with latent syphilis (early or late latent syphilis) have the same testing rate, (4) individuals at the low activity group have lower testing rate than those at high activity group. The $\tau_{i}^{j,NSD}(i\in\{1,2,...,6\},j\in\{L,H\})$ were calculated as:

**1.2 Model simulation of the self-DARE**

We simulated the introduction of regular self-DARE in 2025 and evaluated its impact over a ten-year period (2025–2034). In addition to a base case scenario without self-DARE, we evaluated two intervention strategies: (1) the “only high group” strategy, where self-DARE was recommended exclusively for individuals in the high-activity group presenting for syphilis testing; and (2) the “both groups” strategy, where self-DARE was recommended for all individuals, including those in both high- and low-activity groups, attending for syphilis testing. In these two strategies, the proportions of individuals high activity group and low activity group attending for syphilis testing and participate the self-DARE intervention would be $\chi_{H}$ and $\chi_{L}$.

1. “only high group” strategy: ,
2. “both groups” strategy: ,

where the is the uptake of self-DARE, and we set 72% as its base estimates.

Regular self-DARE was modeled as an additional self-screening approach. Participants were assumed to detect abnormal anorectal findings (including primary syphilis at the anorectum), leading to more frequent clinical visits, testing, diagnosis, and treatment.

Thus, we simulated the impact of self-DARE by incorporating increased rates of syphilis diagnosis and treatment among the participants as follows:

$$\tau_{i}^{j,SD}(t)=\left\{\begin{matrix} \tau_{i}^{j,SD}(t)+\frac{S_{e}\times\alpha\times\varepsilon\times\theta_{1}}{F},(i=1) \\ \tau_{i}^{j,SD}(t)+\frac{\left(1-S_{p}\right)\times\alpha\times\varepsilon\times\theta_{2}}{F},(i\in[2,3,4,5,6]) \end{matrix}\right.$$

In this formula, the $S_{e}$, $S_{p}$, and $F$ denote the sensitivity, specificity, frequency, adherence of self-DARE. We assigned the base estimates of sensitivity and specificity as 60% and 80%, according to previous population study.^4^ Furthermore, we integrated several parameters into the simulation, based on data from a previous 12-week cohort study at the Melbourne Sexual Health Centre involving 30 MSM. In this study, 47% (14/30) of participants consistently performed weekly self-DARE as requested. Throughout the study, participants reported 303 instances of self-DARE, of which 5% (15/303) led to the identification of anorectal abnormalities, and 40% (6/15) of these cases sought clinical review, though none were diagnosed with primary syphilis after serology test. Based on this study, we got base values for three critical parameters in our simulation: (1) the adherence to conducting self-DARE as required $\alpha$ was set at 47%, (2) the proportion of individuals seeking medical review after identifying anorectal abnormalities $\varepsilon$ was set at 40%, and (3) the proportion of MSM without anorectal primary syphilis who exhibited anorectal abnormalities $\theta_{2}$ was set at 5%. Additionally, we assumed a 100% of proportion for anorectal abnormalities among MSM with primary syphilis $\theta_{1}$.

**1.3 Calculation of the key indicators in the model**

1.3.1 Calculation of the calibrated indicators

We calculated the following indicators corresponding to the annual incidence rates and annual testing rates between 2012 and 2022 in a published study.^5^

1. Annual incidence rates:

$$Inci_{year_{j}}^{rate}=\frac{\sum_{i=\left(j-1\right)\times365+1}^{j\times365} (\lambda^{L}(t)\times S^{L,NSD}+\lambda^{H}(t)\times S^{H,NSD})}{N_{L}+N_{H}}$$

1. Annual testing rates:

$$\begin{matrix} Test_{year_{j}}=\sum_{i=\left(j-1\right)\times365+1}^{j\times365} ((\tau_{1}^{L,NSD}(t)I_{1A}^{L,NSD}+\tau_{2}^{L,NSD}(t)I_{1P}^{L,NSD}+\tau_{3}^{L,NSD}(t)(E_{L1}^{L,NSD}+E_{L2}^{L,NSD}+L^{L,NSD})+\tau_{4}^{L,NSD}(t)I_{2}^{L,NSD}+\tau_{5}^{L,NSD}(t)I_{3}^{L,NSD}) \\ +(\tau_{1}^{H,NSD}(t)I_{1A}^{H,NSD}+\tau_{2}^{H,NSD}(t)I_{1P}^{H,NSD}+\tau_{3}^{H,NSD}(t)(E_{L1}^{H,NSD}+E_{L2}^{H,NSD}+L^{H,NSD})+\tau_{4}^{H,NSD}(t)I_{2}^{H,NSD}+\tau_{5}^{H,NSD}(t)I_{3}^{H,NSD}) \\ +\tau_{6}^{L,NSD}(t)(S^{L,NSD}+E^{L,NSD}+T^{L,NSD})+\tau_{6}^{H,NSD}(t)(S^{H,NSD}+E^{H,NSD}+T^{H,NSD}))/(N_{L}+N_{H}) \end{matrix}$$

Based on prior and posterior distributions, we applied the Markov Chain Monte Carlo (MCMC) method to conduct calibration and obtain samples from the joint posterior distribution of parameters. Then we selected 1000 parameter sets for subsequent simulations (**Table S2**).

1.3.2 Calculation of the outcome indicators

We calculated the following outcome indicators in all scenarios to describe the impact and cost-effectiveness in each scenario:

1. Annual incidence cases:

$$Inci_{year_{j}}^{case}=\sum_{i=\left(j-1\right)\times365+1}^{j\times365} (\lambda^{L}(t)\times(S^{L,NSD}+S^{L,SD})+\lambda^{H}(t)\times(S^{H,NSD}+S^{H,SD}))$$

1. Annual stage-specific diagnoses:

$$\begin{matrix} Diag_{I_{1}}^{year_{j}} =\sum_{i=\left(j-1\right)\times365+1}^{j\times365} (\tau_{1}^{L,NSD}(t)I_{1A}^{L,NSD}+\tau_{1}^{H,NSD}(t)I_{1A}^{H,NSD}+\tau_{1}^{L,SD}(t)I_{1A}^{L,SD}+\tau_{1}^{H,SD}(t)I_{1A}^{H,SD} \\ +\tau_{2}^{L,NSD}(t)I_{1P}^{L,NSD}+\tau_{2}^{H,NSD}(t)I_{1P}^{H,NSD}+\tau_{2}^{L,SD}(t)I_{1P}^{L,SD}+\tau_{2}^{H,SD}(t)I_{1P}^{H,SD}) ; \\ Diag_{I_{2}}^{year_{j}} =\sum_{i=\left(j-1\right)\times365+1}^{j\times365} (\tau_{4}^{L,NSD}(t)I_{2}^{L,NSD}+\tau_{4}^{H,NSD}(t)I_{2}^{L,NSD}+\tau_{4}^{L,NSD}(t)I_{2}^{L,NSD}+\tau_{4}^{H,NSD}(t)I_{2}^{H,SD}); \\ Diag_{EL}^{year_{j}} =\sum_{i=\left(j-1\right)\times365+1}^{j\times365} (\tau_{3}^{L,NSD}(t)(E_{L1}^{L,NSD}+E_{L2}^{L,NSD})+\tau_{3}^{H,NSD}(t)(E_{L1}^{H,NSD}+E_{L2}^{H,NSD}) \\ +\tau_{3}^{L,SD}(t)(E_{L1}^{L,SD}+E_{L2}^{L,SD})+\tau_{3}^{H,SD}(t)(E_{L1}^{H,SD}+E_{L2}^{H,SD})); \\ Diag_{Late}^{year_{j}} =\sum_{i=\left(j-1\right)\times365+1}^{j\times365} (\tau_{3}^{L,NSD}(t)L^{L,NSD}+\tau_{5}^{L,NSD}(t)I_{3}^{L,NSD}+\tau_{3}^{H,NSD}(t)L^{H,NSD}+\tau_{5}^{H,NSD}(t)I_{3}^{H,NSD} \\ +\tau_{3}^{L,SD}(t)L^{L,SD}+\tau_{5}^{L,NSD}(t)I_{3}^{L,SD}+\tau_{3}^{H,SD}(t)L^{H,SD}+\tau_{5}^{H,SD}(t)I_{3}^{H,SD}) \end{matrix}$$

1. Quality-adjusted life years (QALY) loss:

$$QALY_{loss}=\sum_{2025}^{2034} QALY_{loss}^{\inf}*\frac{Inci_{year_{j}}^{case}-Inci_{year_{j}}^{case}}{\left(1+\alpha\right)^{j}}$$

where the $QALY_{loss}^{\inf}$ is the average QALY loss of every new syphilis infection among MSM,^6^ and the $\alpha$ represents the annual discount rate.

1. Cost of the self-DARE intervention:

where the $\mathrm{Cos}t_{scenario n}^{Link}$ represents the cost of linking MSM to participate the self-DARE, which was calculated as:

$$\begin{matrix} \mathrm{Cos}t_{year_{j}}^{Link}=\sum_{i=\left(j-1\right)\times365+1}^{j\times365} C_{Link}(\chi_{L}(\tau_{1}^{L,NSD}(t)I_{1A}^{L,NSD}+\tau_{2}^{L,NSD}(t)I_{1P}^{L,NSD}+\tau_{3}^{L,NSD}(t)(E_{L1}^{L,NSD}+E_{L2}^{L,NSD}+L^{L,NSD}) \\ +\tau_{4}^{L,NSD}(t)I_{2}^{L,NSD}+\tau_{5}^{L,NSD}(t)I_{3}^{L,NSD}+\tau_{6}^{L,NSD}(t)(S^{L,NSD}+E^{L,NSD}+T^{L,NSD})) \\ +\chi_{H}(\tau_{1}^{H,NSD}(t)I_{1A}^{H,NSD}+\tau_{2}^{H,NSD}(t)I_{1P}^{H,NSD}+\tau_{3}^{H,NSD}(t)(E_{L1}^{H,NSD}+E_{L2}^{H,NSD}+L^{H,NSD}) \\ +\tau_{4}^{L,NSD}(t)I_{2}^{L,NSD}+\tau_{5}^{L,NSD}(t)I_{3}^{L,NSD}+\tau_{6}^{L,NSD}(t)(S^{L,NSD}+E^{L,NSD}+T^{L,NSD}))) \end{matrix}$$

The $\mathrm{Cos}t_{year_{j}}^{ExtraSDT}$ represents the annual cost of extra screening, diagnosing, and treatment due to self-DARE, which was calculated as:

$$\begin{matrix} Cost_{year_{j}}^{ExtraSDT}=\sum_{i=\left(j-1\right)\times365+1}^{j\times365} \{Cost_{screen}[(\tau_{6}^{H,SD}(t)-\tau_{6}^{H,NSD}(t))(S^{H,SD}+E^{H,SD}+T^{H,SD})(\tau_{6}^{L,SD}(t)-\tau_{6}^{L,NSD}(t))(S^{L,SD}+E^{L,SD}+T^{L,SD})] \\ +Cost_{early}[(\tau_{1}^{H,SD}(t)-\tau_{1}^{H,NSD}(t))I_{1A}^{H,SD}+(\tau_{2}^{H,SD}(t)-\tau_{2}^{H,NSD}(t))I_{1P}^{H,SD}+(\tau_{3}^{H,SD}(t)-\tau_{3}^{H,NSD}(t))(E_{L1}^{H,SD}+E_{L2}^{H,SD}) \\ +(\tau_{4}^{H,SD}(t)-\tau_{4}^{H,NSD}(t))I_{2}^{H,SD}+(\tau_{1}^{L,SD}(t)-\tau_{1}^{L,NSD}(t))I_{1A}^{L,SD}+(\tau_{2}^{L,SD}(t)-\tau_{2}^{L,NSD}(t))I_{1P}^{L,SD} \\ +(\tau_{3}^{H,SD}(t)-\tau_{3}^{H,NSD}(t))(E_{L1}^{H,SD}+E_{L2}^{H,SD})+(\tau_{4}^{H,SD}(t)-\tau_{4}^{H,NSD}(t))I_{2}^{H,SD}] \\ +Cost_{late}[(\tau_{3}^{H,SD}(t)-\tau_{3}^{H,NSD}(t))L^{H,SD}+(\tau_{5}^{H,SD}(t)-\tau_{5}^{H,NSD}(t))I_{3}^{H,SD}+(\tau_{3}^{L,SD}(t)-\tau_{3}^{L,NSD}(t))L^{L,SD} \\ +(\tau_{5}^{L,SD}(t)-\tau_{5}^{L,NSD}(t))I_{3}^{L,SD})]\} \end{matrix}$$

where the costs were calculated as:

$$\begin{matrix} Cost_{screen}=C_{GP-B}+C_{SeroTest-1} \\ Cost_{early}=C_{GP-B}+C_{SeroTest-1}+ C_{GP-C}+C_{SeroTest-2}+C_{NAAT-1}+C_{GP-B}+C_{SeroTest-1}+C_{BPG} \\ Cost_{late}=C_{GP-B}+C_{SeroTest-1}+ C_{GP-C}+C_{SeroTest-2}+C_{NAAT-1}+C_{GP-B}+\left(C_{SeroTest-1}+C_{BPG}\right)\times3 \end{matrix}$$

1. Incremental cost-effectiveness ratio (ICER) of self-DARE

$$ICER=\frac{QALY_{loss}^{base case}-QALY_{loss}^{self-DARE}}{Cost_{self-DARE}^{Increase}}$$

where the $Cost_{self-DARE}^{Increase}$ represents the increase of total cost of self-DARE scenarios compared with the base case, and was calculated as:

$$\begin{matrix} Cost_{self-DARE}^{Increase}=\sum_{2025}^{2034} (Cost_{screen}(Screen_{self-DARE}-Screen_{base case})+Cost_{early}(Diag_{self-DARE}^{early syphilis}-Diag_{base case}^{early syphilis}) \\ +Cost_{late}(Diag_{self-DARE}^{late syphilis}-Diag_{base case}^{late syphilis})+\mathrm{Cos}t_{year_{j}}^{Link})/(1+\alpha)^{j} \end{matrix}$$

1. Cost per infection averted:

1. Benefit cost ratio (BCR)

$$\begin{matrix} BCR=(\sum_{2025}^{2034} (Cost_{screen}Screen_{base case}+Cost_{early}Diag_{base case}^{early syphilis}+Diag_{base case}^{late syphilis})/(1+\alpha)^{j}- \\ (\sum_{2025}^{2034} (Cost_{screen}Screen_{self-DARE}+Cost_{early}Diag_{self-DARE}^{early syphilis}+Cost_{late}Diag_{self-DARE}^{late syphilis})/(1+\alpha)^{j} \\ -Cost_{self-DARE}))/Cost_{self-DARE} \end{matrix}$$

**Table S1 The values of transmission model parameters derived from references.**

| Parameter | Description | Value (ranges) | Unit | Source |
| --- | --- | --- | --- | --- |
| $N$ | Population size of GBMSM in Australia | 130,000 | - | 7 |
| $\nu$ | Proportion of GBMSM in high sexual activity | 80 | % | 8 |
| $\frac{1}{\rho_{1}}$ | The mean time from the exposed stage to primary syphilis | 24.5 | days | 9 |
| $\frac{1}{\rho_{2}}$ | The mean time from the primary syphilis stage to second early latent syphilis | 52.5 | days | 9 |
| $\frac{1}{\rho_{3}}$ | The mean time from the second early latent syphilis stage to secondary syphilis | 63 | days | 10 |
| $\frac{1}{\rho_{4}}$ | The mean time from the secondary syphilis stage to third early latent syphilis | 120 | days |  |
| $\frac{1}{\rho_{5}}$ | The mean time from the third early latent syphilis stage to late latent syphilis | 343 | days |  |
| $\frac{1}{\rho_{6}}$ | The mean time from the late latent syphilis stage to tertiary syphilis | 15 | years |  |
|  | The mean duration of the immunity of late syphilis after treatment | 5 | years |  |
| $\alpha$ | Adherence of participants conducting the self-DARE as required. | 47% (10-100%) | - | 11 |
| $\varepsilon$ | Proportion of participants seeking for clinical review after detecting anorectal abnormalities. | 40% (10-100%) | - | 11 |
| $\theta_{1}$ | Proportion of anorectal abnormalities in individuals with anorectal primary syphilis. | 100% (10-100%) | - | Assumed |
| $\theta_{2}$ | Proportion of anorectal abnormalities except primary syphilis | 5% (0-10%) | - | 11 |
| $S_{e}$ | Sensitivity of self-DARE | 60% (10-100%) | - | 4 |
| $S_{p}$ | Specificity of self-DARE | 80% (10-100%) | - | 4 |
|  | Uptake of self-DARE recommendation | 72% (35-98%) | - | 12 |
| $\delta$ | Duration of participants keeping conducted self-DARE | 180 (90-360) | days | Assumed based on ^12^ |
| $F$ | Frequency of participants conducted self-DARE | 30 (7-90) | Once days^-1^ | Assumed based on ^11,12^ |

**Table S2 Prior distributions and posterior estimates of uncertain parameters**

| Parameter | Description | Prior distributions | Source | Posterior estimates  (Median and 95% CrI) |
| --- | --- | --- | --- | --- |
| $\eta$ | Proportion of syphilis progress to anorectal primary syphilis | U[0,1] | - | 0.553 (0.220-0.839) |
| $\beta_{1,0}$ | Initial per-partner transmission rate of anorectal primary syphilis | U[0,1] | - | 0.480 (0.124-0.883) |
| $\beta_{2,0}$ | Initial per-partner transmission rate of penile primary syphilis | U[0,1] | - | 0.616 (0.040-0.988) |
| $\beta_{3,0}$ | Initial per-partner transmission rate of early latent syphilis | U[0,1] | - | 0.410 (0.159-0.921) |
| $\beta_{4,0}$ | Initial per-partner transmission rate of secondary syphilis | U[0,1] | - | 0.443 (0.075-0.946) |
| $\tau_{1,0}$ | Initial daily testing rates of anorectal primary syphilis | U[0,0.1] | Assumed | 0.006 (0.001-0.014) |
| $\tau_{2,0}$ | Initial daily testing rates of penile primary syphilis | U[0,0.1] | Assumed | 0.055 (0.019-0.099) |
| $\tau_{3,0}$ | Initial daily testing rates of latent syphilis | U[0,0.1] | Assumed | 0.006 (0.001-0.014) |
| $\tau_{4,0}$ | Initial daily testing rates of secondary syphilis | U[0,0.1] | Assumed | 0.061 (0.023-0.098) |
| $\tau_{5,0}$ | Initial daily testing rates of tertiary syphilis | U[0,0.1] | Assumed | 0.065 (0.002-0.094) |
| $\tau_{6,0}$ | Initial daily testing rates of susceptible, exposed, and immune individuals | U[0,0.1] | Assumed | 0.005 (0.004-0.006) |
| $\theta_{\beta}$ | Annual increase in transmission risk behavior | U[0,1] | - | 0.068 (0.035-0.112) |
| $\theta_{\tau}$ | Annual increase in testing rate | U[0,1] | - | 0.122 (0.115-0.131) |
| $\mu_{L}$ | Annual rate of partner change in group L | U[0,10] | 8 | 1.269 (0.564-2.586) |
| $\mu_{H}$ | Annual rate of partner change in group H | U[10,20] | 8 | 13.364 (10.134-17.467) |
| $\zeta$ | Level of assortativity in sexual mixing | U[0,1] | - | 0.498 (0.071-0.907) |
| $\omega$ | Ratio of testing rate in group L vs H | U[0,1] | - | 0.168 (0.102-0.288) |
| $P_{L}$ | Initial prevalence of exposed infection in group H | U[0, 0.01] | Assumed | 0.009 (0.003-0.015) |
| $P_{H}$ | Initial prevalence of exposed infection in group H | U[0,0.1] | Assumed | 0.014 (0.004-0.022) |

**Table S3 The economic parameters values for health-economic analysis**

| Parameter | Description | Value | Range | Unit | Source |
| --- | --- | --- | --- | --- | --- |
|  | Cost of general practitioner of level B | 42.9 | - | 2024 A$ | 13 |
|  | Cost of general practitioner of level C | 82.9 | - | 2024 A$ | 13 |
|  | Cost of single serology test for syphilis | 15.7 | - | 2024 A$ | 13 |
|  | Cost of double serology test for syphilis | 29.0 | - | 2024 A$ | 13 |
|  | Cost of single nucleic acid amplification test (NAAT) for syphilis | 28.7 | - | 2024 A$ | 13 |
|  | Cost of the benzathine benzylpenicillin for single time treatment | 67.2 | - | 2024 A$ | 14 |
|  | Cost of recruiting one MSM to participate the self-DARE intervention | 50 | 0-100 | 2024 A$ | 11 |
| $QALY_{loss}$ | Average QALY loss of one syphilis infection in MSM population | 0.06 | - | - | 6 |
|  | Willing-to-pay threshold in the Australia | 50,000 | - | 2024 A$ | 15 |
| $\alpha$ | Annual discount rate | 3% | 0-6% | - | 15 |


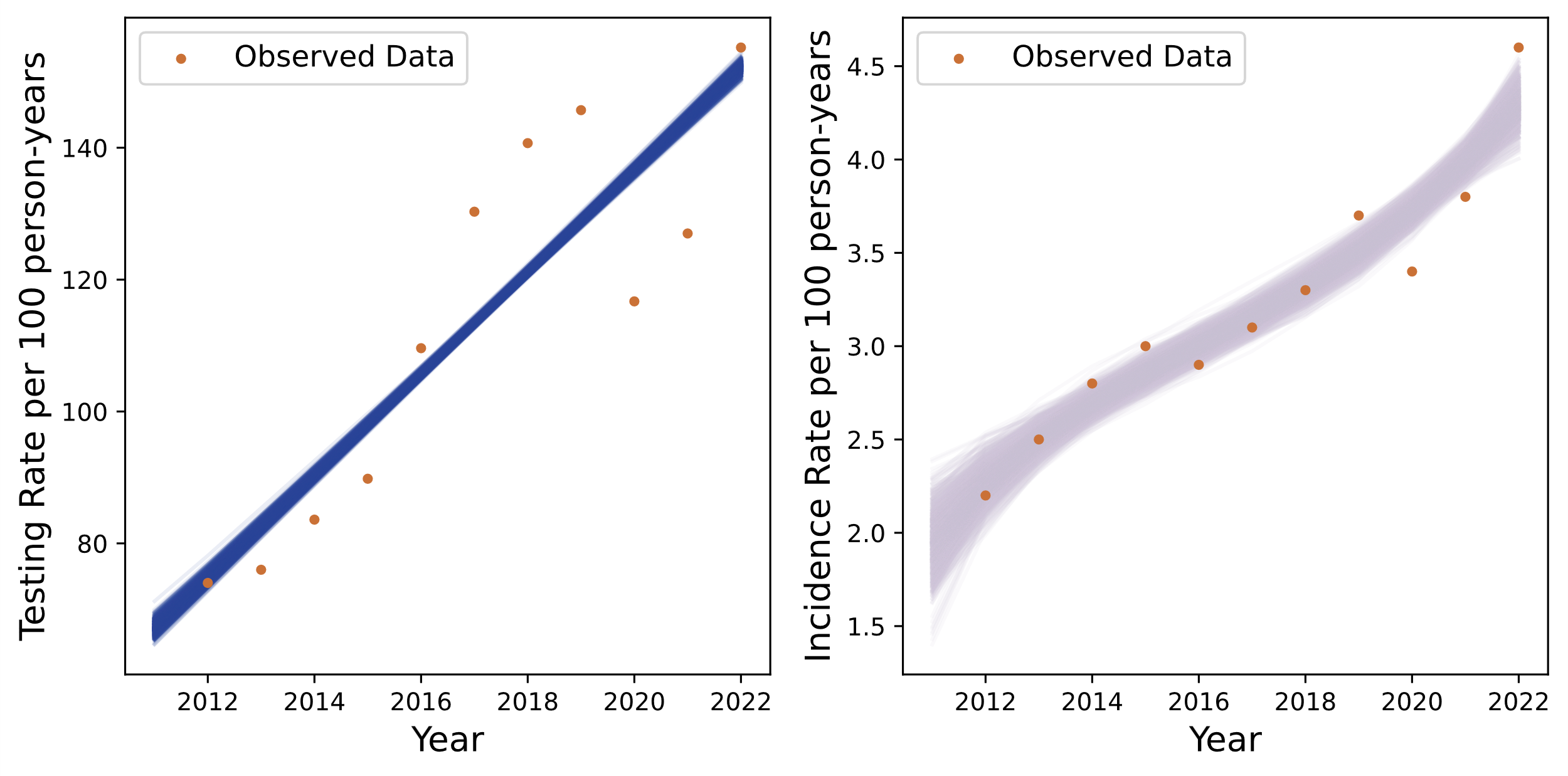


**Figure S1. Calibration results of the model.** This model was calibrated with annual rates of testing and infectious syphilis incidence from 2012 to 2022 among Australian GBMSM were derived from published studies based on the Australian Collaboration for Coordinated Enhanced Sentinel Surveillance (ACCESS) system.^5^ The orange points represent the data estimated, and the lines represent the corresponding outcomes in the simulations with all parameter sets.


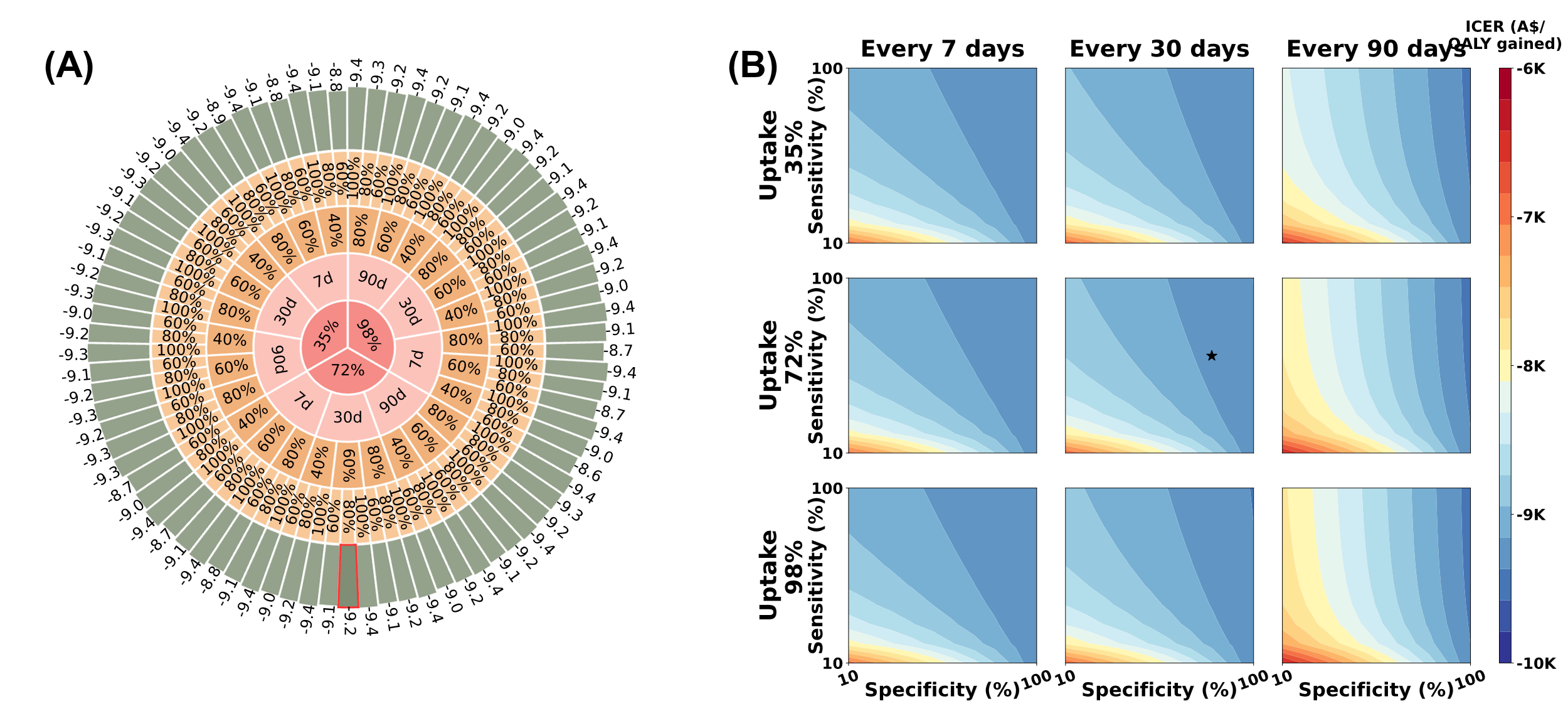


**Figure S2. Sensitivity analysis of the ICERs in scenarios providing self-DARE for high activity GBMSM. (A)-(B).** The bar with red frame in panels (A) and the pentagrams in panels (B) represent the outcomes in “only high group” scenario with base estimates of the features.


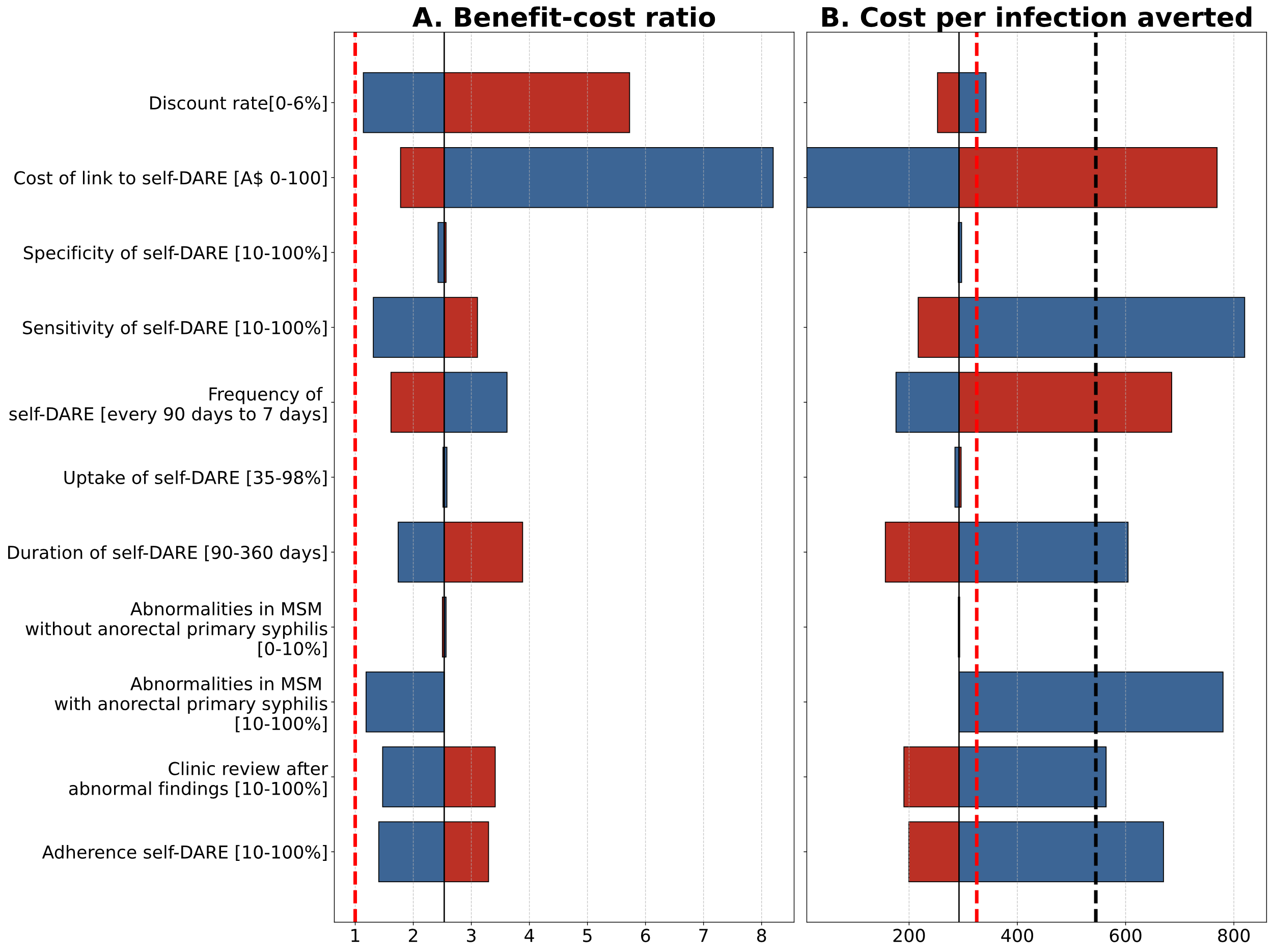


**Figure S3. One-way sensitivity analysis of the costs per infection averted and benefit-cost ratios in scenarios only providing self-DARE for high activity GBMSM.** The horizontal bars represent the range of cost per infection averted (cost per IA), and benefit cost ratio (BCR) of self-DARE, as each parameter varies across their plausible range. The solid vertical black lines indicate the outcomes’ values under base estimates of these parameters. The red dashed line in panel A represents the threshold of 1. The red and black dashed lines in panel B represent the total cost of diagnosing and treating early syphilis (AUD $325) and late syphilis (AUD $545), respectively.


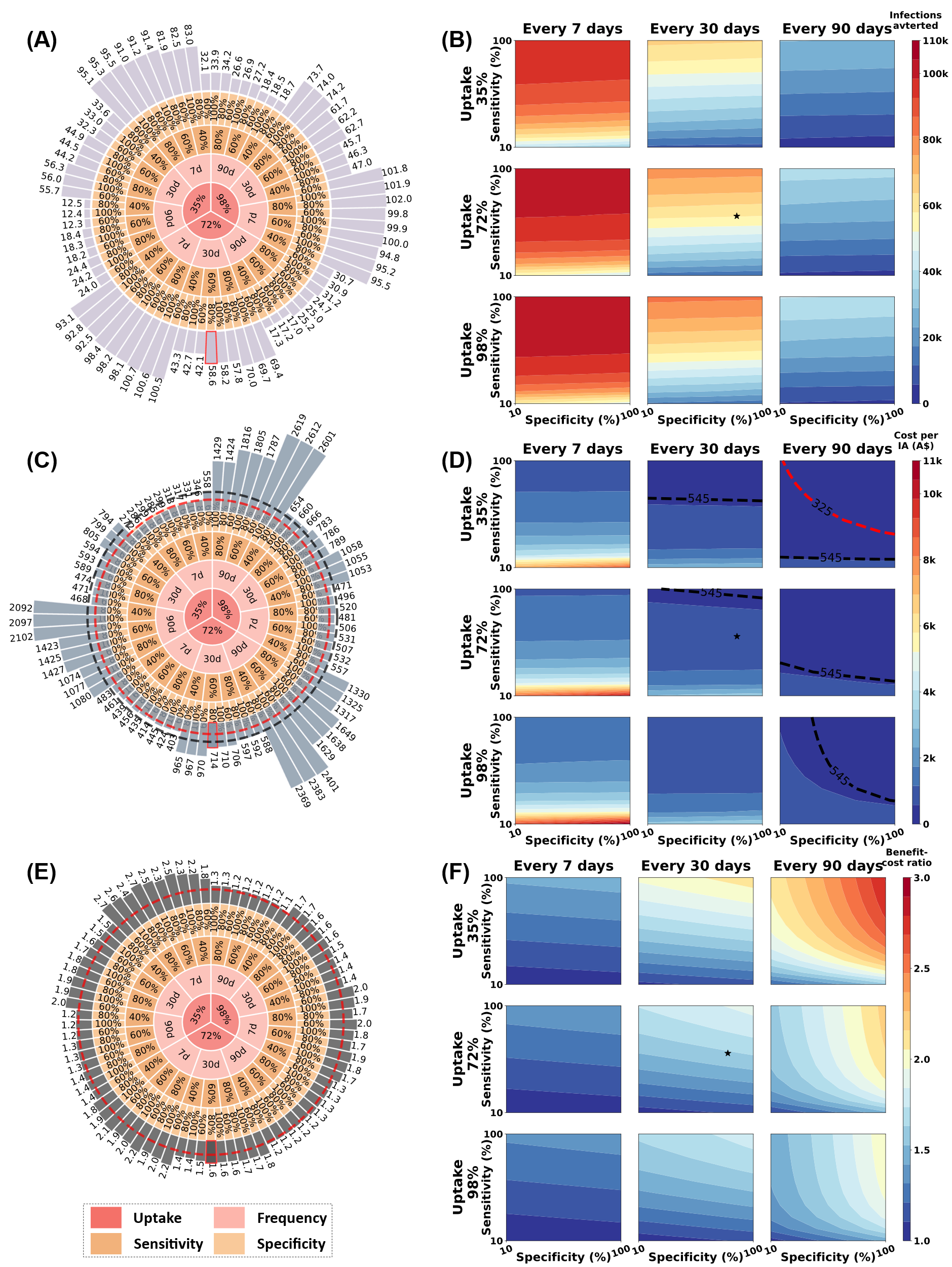


**Figure S4. Sensitivity analysis of the epidemiological and health-economical outcomes in scenarios only providing self-DARE for all GBMSM. (A)-(B).** The number of cumulative infections averted between 2025 to 2034; **(C)-(D).** The cost of per infection averted by implementing self-DARE, with red and black dashed lines representing the costs of diagnosing and treating one early syphilis case (AUD $325) and one late syphilis case (AUD $545), respectively; **(E)-(F).** The benefit-cost ratios, with red dashed line indicating a benefit-cost ratio of one. The bars with red frames in panels (A), (C), and (E), and the pentagrams in panels (B), (D), and (F) represent the outcomes in “base groups” scenario with base estimates of the features.


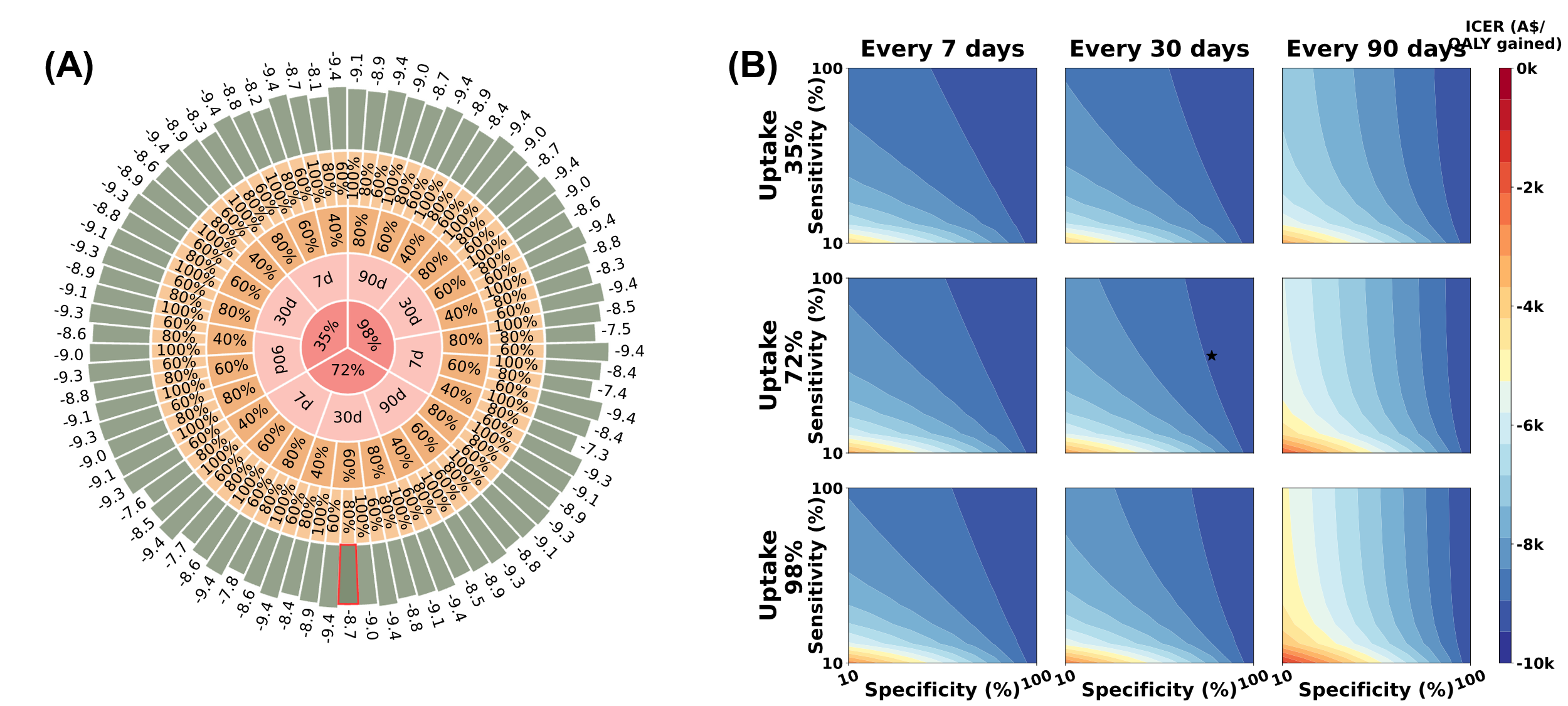


**Figure S5. Sensitivity analysis of the ICERs in scenarios providing self-DARE for all GBMSM. (A)-(B).** The bar with red frame in panels (A) and the pentagrams in panels (B) represent the outcomes in “only high group” scenario with base estimates of the features.

**References**

1 Hui BB, Ward JS, Guy R, Law MG, Gray RT, Regan DG. Impact of Testing Strategies to Combat a Major Syphilis Outbreak Among Australian Aboriginal and Torres Strait Islander Peoples: A Mathematical Modeling Study. *Open Forum Infect Di* 2022; **9**. DOI:10.1093/ofid/ofac119.

2 Peeling RW, Mabey D, Kamb ML, Chen X-S, Radolf JD, Benzaken AS. Syphilis. *Nat Rev Dis Primers* 2017; **3**. DOI:10.1038/nrdp.2017.73.

3 Whittles LK, Didelot X, White PJ. Public health impact and cost-effectiveness of gonorrhoea vaccination: an integrated transmission-dynamic health-economic modelling analysis. *Lancet Infect Dis* 2022; **22**: 1030–41.

4 Nyitray AG, McAuliffe TL, Liebert C, *et al.* The accuracy of anal self- and companion exams among sexual minority men and transgender women: a prospective analysis. *The Lancet Regional Health - Americas* 2024; **31**: 100704.

5 Traeger MW, Guy R, Taunton C, *et al.* Syphilis testing, incidence, and reinfection among gay and bisexual men in Australia over a decade spanning HIV PrEP implementation: an analysis of surveillance data from 2012 to 2022. *Lancet Reg Health – West Pac* 2024; **51**: 101175.

6 Lee K, You S, Li Y, *et al.* Estimation of the Lifetime Quality-Adjusted Life Years (QALYs) Lost Due to Syphilis Acquired in the United States in 2018. *Clin Infect Dis* 2023; **76**: e810–9.

7 Callander D, Mooney-Somers J, Keen P, *et al.* Australian ‘gayborhoods’ and ‘lesborhoods’: a new method for estimating the number and prevalence of adult gay men and lesbian women living in each Australian postcode. *International Journal of Geographical Information Science* 2020; **34**: 2160–76.

8 Annual Report of Trends in Behaviour 2024. UNSW Sites. https://unsworks.unsw.edu.au/bitstreams/a882159e-b8ef-4b0b-9d63-ba0a2b579f8b/download (accessed Aug 28, 2024).

9 Hoare A, Gray RT, Wilson DP. Could implementation of Australia’s National Gay Men’s Syphilis Action Plan have an indirect effect on the HIV epidemic? *Sex Health* 2012; **9**: 144.

10 French P. Syphilis. *BMJ* 2007; **334**: 143–7.

11 Aung ET, Fairley CK, Ong JJ, *et al.* Adherence to weekly anal self-examination among men who have sex with men for detection of anal syphilis. *Front Med* 2022; **9**: 941041.

12 Aung ET, Chow EPF, Fairley CK, *et al.* Preferences of men who have sex with men for performing anal self-examination for the detection of anal syphilis in Australia: A discrete choice experiment. *The Lancet Regional Health - Western Pacific* 2022; **21**: 100401.

13 Australian Government—Department of Health and Aged Care. MBS online. https://www.mbsonline.gov.au/internet/mbsonline/publishing.nsf/Content/Downloads-240301 (accessed March 6, 2024).

14 Australian Government—Department of Health and Aged Care. The Pharmaceutical Benefits Scheme. https://www.pbs.gov.au/browse/downloads (accessed March 6, 2024).

15 Lew J-B, John DJBS, Xu X-M, *et al.* Long-term evaluation of benefits, harms, and cost-effectiveness of the National Bowel Cancer Screening Program in Australia: a modelling study. *The Lancet Public Health* 2017; **2**: e331–40.
